# Private anomaly detection of student health conditions based on wearable sensors in mobile cloud computing

**DOI:** 10.1186/s13677-022-00300-x

**Published:** 2022-09-05

**Authors:** Yu Xie, Kuilin Zhang, Huaizhen Kou, Mohammad Jafar Mokarram

**Affiliations:** 1 Chengdu University of Information Technology, Chengdu, China; 2 Shandong Provincial University Laboratory for Protected Horticulture, Weifang University of Science and Technology, Weifang, China; 3 School of Computer Science and Engineering, Nanjing University of Science and Technology, Nanjing, China; 4 Department of Electrical and Electronics Engineering, Shiraz University of Technology, Shiraz, Iran

**Keywords:** Mobile cloud computing, Healthcare, Privacy, Wearable sensors, Anomaly detection

## Abstract

With the continuous spread of COVID-19 virus, how to guarantee the healthy living of people especially the students who are of relative weak physique is becoming a key research issue of significant values. Specifically, precise recognition of the anomaly in student health conditions is beneficial to the quick discovery of potential patients. However, there are so many students in each school that the education managers cannot know about the health conditions of students in a real-time manner and accurately recognize the possible anomaly among students quickly. Fortunately, the quick development of mobile cloud computing technologies and wearable sensors has provided a promising way to monitor the real-time health conditions of students and find out the anomalies timely. However, two challenges are present in the above anomaly detection issue. First, the health data monitored by massive wearable sensors are often massive and updated frequently, which probably leads to high sensor-cloud transmission cost for anomaly detection. Second, the health data of students are often sensitive enough, which probably impedes the integration of health data in cloud environment even renders the health data-based anomaly detection infeasible. In view of these challenges, we propose a time-efficient and privacy-aware anomaly detection solution for students with wearable sensors in mobile cloud computing environment. At last, we validate the effectiveness and efficiency of our work via a set of simulated experiments.

## Introduction

Currently, the wide spread of COVID-19 virus is a huge threat for the healthy living and life of people all over the world. Affected by the pandemic, people are more caring about their personal health conditions than ever before [1–3]. Especially for the students who are often of relatively weaker physique compared with adults, more attentions should be paid to know about the health conditions of students effectively and timely. Generally, through observing the health data of students, we can mine, analyze and discover the possible exceptions or anomalies existing in candidate students. Such anomalies can help education managers or medical departments to quickly filter out the possible patients [4–6].

However, the above anomaly detection process is often non-trivial because there are so many students in each school and the physique of each student is often varied. Fortunately, the quick development of mobile cloud computing technologies and wearable sensors has provided a promising way for school managers to know about the health conditions of students and find out the prospective anomalies timely [7–9]. For example, popular mobile devices (e.g., mobile phones, smart watches, wearable sensors, etc) can monitor the health conditions (e.g., blood pressure, heart rate and so on) of students in a real-time way [10] and transmitted them to a remote cloud platform. Through analyze the mine such monitored health data in cloud, we can get to know the real-time health conditions of students and filter out the possible patients from candidate students through anomaly detection technologies [11–13].

However, two challenges are often present in the above anomaly detection process. First, the health data monitored by massive wearable sensors of students are often massive and updated frequently with time elapsing [14–16], which probably brings high sensor-cloud data transmission cost for anomaly detection. Second, the health data of students are often sensitive enough, which probably impedes the integration of health data in cloud especially when there are no enough and attractive incentive mechanisms [17, 18]. In this situation, the data sparsity probably renders the health data-based anomaly detection infeasible. In view of these challenges, we propose a time-efficient and privacy-aware anomaly detection solution for students with wearable sensors in mobile cloud computing environment. Concretely, we adopt an effective privacy-preserving technique to guarantee the sensitive information of people is secure, which can minimize the privacy disclosure concerns of people when a cloud platform integrates the distributed data of people together for uniform data processing and mining. At last, we validate the effectiveness and efficiency of our work via a set of simulated experiments.

In summary, the major novelty or contributions in this work are as follows.

1. We recognize the significance of anomaly detection in accurate recognition of potential patients, and realize the importance of time-aware health data monitored by wearable sensors in anomaly detection.
2. We propose an anomaly detection method for student health conditions based on wearable sensors in mobile cloud computing, named Ano-Det. The proposal can achieve efficient and privacy-aware anomaly detection.
3. Simulated experiments are enacted and deployed to prove the feasibility of the proposal in terms of anomaly detection performances including accuracy, privacy-preservation and computational time in cloud environment. In Ano-Det, we use a hash technique to achieve the two goals of data efficiency and data privacy simultaneously since (a) hash indexes can be created offline whose time complexity is approximately O(1) and (b) hash indexes can secure the sensitive information of students well.

We summarize the rest of paper as follows. Section 2 reviews the state-of-the-art literature in the field. A motivating example is constructed in Section 3 to clarify the research background and challenges focused in this paper. Concrete steps of our proposed Ano-Det method are described in Section 4. Evaluation is made in Section 5. At last, conclusions are drawn in Section 6.

## Related work

We investigate the current research literature about anomaly detection in health domain as follows.

### Anomaly detection in big data

One of the major characteristics of big data is that the data are coarse [19–22] and therefore, pre-processing treasure before transmitting the data to a cloud platform is necessary to cope with the probably existed anomaly points. As an important part of civil infrastructure, health monitoring system generates a large amount of data; but there are many noises in the data, which is very time-consuming to detect. To tackle the above problem, the authors in [23] propose a data anomaly detection method based on computer vision and deep learning. Overall, the method can be divided into two stages. Firstly, data is converted through visualization, and the construction process of abnormal classification neural network is carried out at the same time. In the second stage, randomly selected training data are input into the neural network for training. After in-depth training, anomalies in a big volume of data can be detected. In [24], anomaly detection in data pre-processing stage before sensor-cloud data transmission is firstly studied, and a new data anomaly detection method based on convolutional neural network is proposed, which mainly imitates human visual and decision-making behavior. Experiments show that the proposed method can detect the abnormal condition of structural health data accurately and efficiently. In [25], an anomaly detection method is proposed combining CNN and GRU. In this method, the stacked convolutional neural network layer is used to capture the input data and extract the features, and then the stacked gated cyclic unit is used to learn the time features. Finally, anomaly detection is performed in the regression layer.

In [26], the authors present a new anomaly detection method which combines the Bayesian dynamic linear model with the switched Kalman filter theory. This method is based on the prior probability of abnormal state and the transition probability between normal state and abnormal state. Importantly, this approach operates under semi-supervised conditions, where normal and abnormal state labels do not require training models. As an unsupervised learning method for anomaly detection, Markov square distance (MSD) has some limitations. Inspired by this observation, work [27] proposes an anomaly detection method for structured health data (SHM) based on adaptive Mahalanobis distance and KNN rules, i.e., AMSD-kNN. The AMSD-kNN method is mainly used to find the nearest neighbor of training set and test set in two steps, to eliminate the estimation of environment change and local covariance matrix. This method provides an unsupervised learning method for SHM through a new distance measure and kNN rules in cloud. In [28], a kind of hash-based anomaly detection method is introduced to recognize the abnormal points in time-related data stream in Internet of Things. Since hash is very efficient in time cost, the recognition speed of anomaly detection is improved significantly especially in the big data context. Other related literature includes [29] where transfer learning technique is adopted to achieve anomaly detection in big data system and [30] where the anomaly detection effect and performances are validated by mathematical way.

However, the above anomaly detection solutions often fall short in protecting the sensitive information of users, which constrains the wide applications of anomaly detection in various big data applications.

### Privacy-aware data utilization

How to secure the sensitive information contained in big data is a key to make full use of the value hidden in big data [31–33]. Hash technique is recruited in [34, 35] to secure the personal information contained in user big data. Concretely, user data are modeled into less-sensitive user indexes and then the user indexes stored in cloud platforms are used in user clustering and missing value prediction. Since user indexes are built offline beforehand, the indexes-based user clustering is very efficient. However, anomaly detection issue is not considered. Blockchain is adopted in [36, 37] to realize the protection of user privacy during the cross-platform data sharing and integration process. This way, the sensitive data stored in different cloud platform can be fully shared for value-added smart applications while guaranteeing not to disclose much private information of users. The advantage of blockchain-based privacy protection solutions is that they are with strong mathematical foundation and confidential degree.

An edge computing-based data sharing and integration method is proposed in [38] to overcome the shortcoming of traditional central big data integration manner according to which user data are transmitted to a remote cloud platform and user information is probably disclosed during data transmission. Differential Privacy technique has been proven an effective privacy protection solution in big data application systems. For example, in [39], the authors use differential privacy to realize the secure sharing of graph data owned by different stakeholders. This way, data owners are willing to publish their respective graph data without the concerns of possible privacy disclosure. Since not all data are useful for creating a big data system, it is not necessary to open all the data owned by users to the public in data sharing. Inspired by this motivation, the authors in [40] put forward a sampling-based data sharing and publishing method. In concrete, only a small portion of user-related data are selected through a sampling process and released to the third party to realize effective data sharing and utilization. This way, most data that are not selected through the sampling process are secured well. In addition, [41] proposed a federated learning method for protecting user sensitive data in the Internet of Things scenario, in which the POI recommendation problem is solved in parallel with the federated learning framework in distributed systems.

However, the above privacy protection solutions do not consider the anomaly points probably existed in big data applications. Therefore, they cannot address the anomaly detection issue in mobile cloud environment well.

**Fig. 1:**
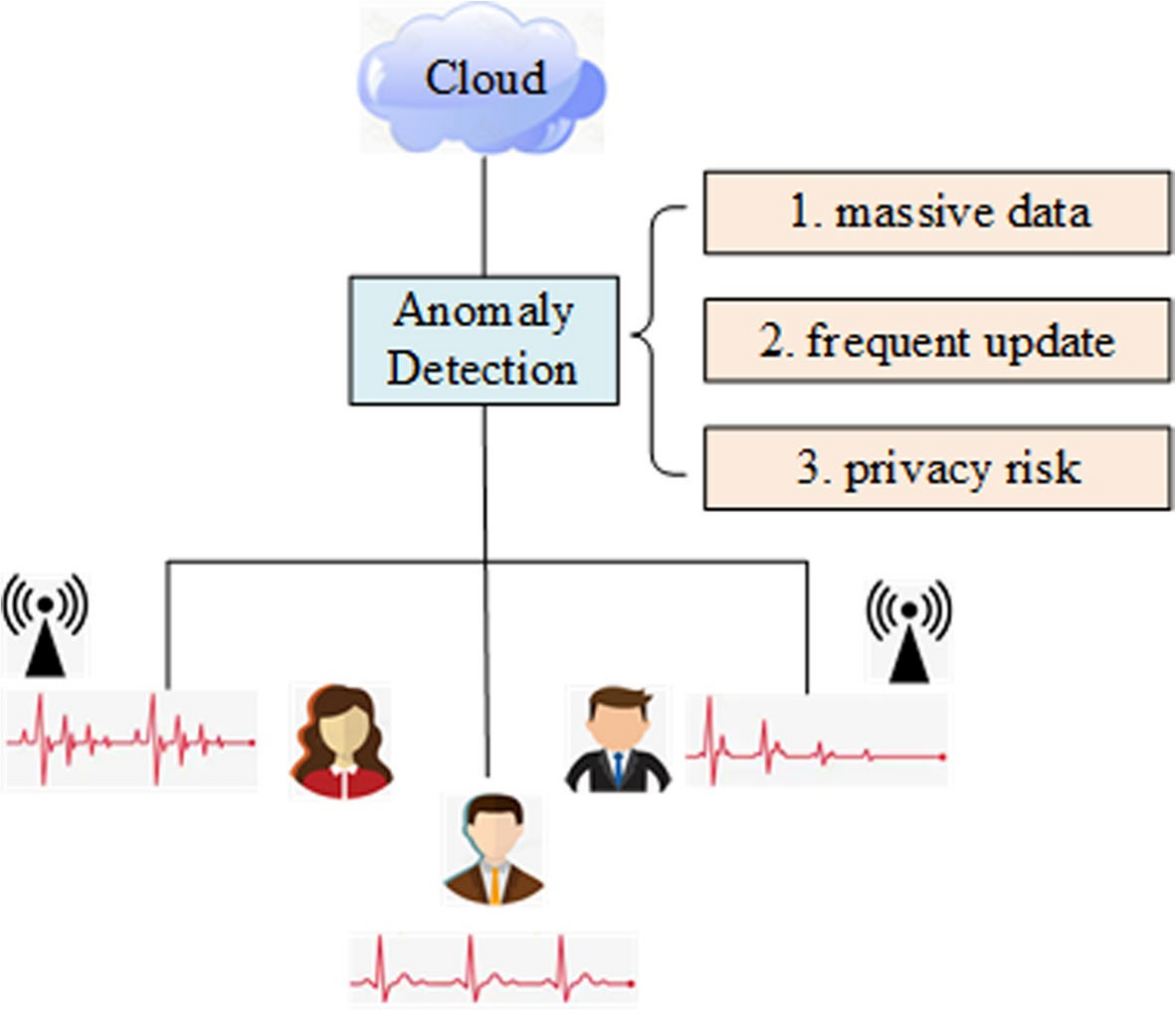
Anomaly detection of health data with wearable sensors in mobile cloud computing: challenges

## Motivation

To better ease the understanding of our motivation, a concrete example is shown in Fig. 1. Here, the health conditions of students are monitored by wearable sensors embedded in various mobile devices (e.g., smart watches, mobile phones, etc.) [42–44]. Thus, we can obtain real-time monitoring data (e.g., electrocardiogram) which can be analyzed and clustered to discover the possible patients from all candidate students. Generally, the monitored health data by wearable sensors need to be transmitted to a remote cloud platform for uniform data processing, during which several challenges are often present. First, the health data monitored by wearable sensors of students are of massive and therefore, the response time of anomaly detection is often long. Second, the students’ health conditions are often varied or updated with time, which requires additional time cost to achieve scalable anomaly detection. Third, the monitored health data by wearable sensors need to be first transmitted to the central cloud platform, during which transmission process user privacy is probably disclosed to malicious third parties. Therefore, user privacy is often at risk in centralized anomaly detection process.

Considering the above three challenges, it is necessary to develop a novel anomaly detection approach for people’s health conditions based on the monitored health data by wearable sensors in mobile cloud computing environment. Therefore, we propose a new Ano-Det method in the following sections to guarantee efficient, scalable and privacy-preserving anomaly detection in big data context.

## Anomaly detection method: Ano-Det

The basic idea of the proposed Ano-Det method is introduced as follows: we firstly convert the health data of students into lightweight health indexes and stored them in the cloud platform; next, we calculate the similarity between each pair of the health conditions of students based on the health indexes; finally, we cluster the students based on their health indexes and discover the possible anomalies based on the clustering results. The concrete details of Ano-Det method is described as follows.

### Step 1: Generate each student’s health index

As indicated in the example in Fig. 1, the students’ health data monitored by wearable sensors are often expressed with a curve which fluctuates with time. Therefore, we first model the students’ health data with a multi-dimensional matrix $\kappa$ depicted in Eqs. (1)-(2). Here, we assume that there are *N* students, i.e., $s_{1}$,..., $s_{N}$ and *M* health criteria (e.g., heart rate, blood pressure, etc), i.e., $c_{1}$,..., $c_{M}$. Moreover, each entry in matrix $\kappa$, i.e., $A_{i,j}$ (*i* = 1, 2,..., *N*; *j* = 1, 2,..., *M*) represents the student $s_{i}$’s health data over criterion $c_{j}$. Furthermore, as described in Fig. 1, each entry $A_{i,j}$ is a time-aware fluctuant curve; therefore, we formulate $A_{i,j}$ with a vector in Eq. (2) where *K* denotes the number of time points at which wearable sensors monitor and record the health conditions of students. For example, *K* = 3 means that three pieces of health data are monitored by wearable sensors. From certain points of view, parameter *K* describes the health data monitoring frequency.

1 $$\begin{aligned} \kappa = \begin{array}{cc} &{} c_{1}\quad \cdots \quad c_{M}\\ \begin{array}{c} s_{1} \\ \vdots \\ s_{N} \end{array} &{} \left[ \begin{array}{ccc} A_{1, 1} &{} \cdots &{} A_{1, M} \\ \vdots &{} \ddots &{} \vdots \\ A_{N, 1} &{} \cdots &{} A_{N, M} \end{array}\right] \end{array} \end{aligned}$$

2 $$\begin{aligned} A_{i, j} = (a_{i, j, 1},\ldots , a_{i, j, K}) \end{aligned}$$

As Eqs. (1)-(2) shows, $$\kappa$$ is an $$N*M*K$$ tensor. To ease the following calculations, we need to convert the $$N*M*K$$ tensor $$\kappa$$ into a multi-dimensional vector. To achieve this goal, we first convert the *K*-dimensional vector $$A_{i,j}$$ into a concrete value. Concretely, we first produce a *K*-dimensional vector *B* presented in Eq. (3). Here, each entry in vector *B* is generated by Eq. (4) where function $$\varGamma (-1, 1)$$ is responsible for producing a random data belonging to [-1, 1]. Thus, with the *K*-dimensional vector $$A_{i,j}$$ and the *K*-dimensional vector *B*, we compute their inner product according to Eq. (5) and the final result is denoted by $$\varOmega _{i, j}$$.

3 $$\begin{aligned} B=(b_{1},\ldots , b_{K}) \end{aligned}$$

4 $$\begin{aligned} b_{k} = \varGamma (-1, 1)(k=1, 2,\ldots , K) \end{aligned}$$

5 $$\begin{aligned} \varOmega _{i, j} = A_{i, j}*B \end{aligned}$$

According to Eq. (5), $\varOmega _{i, j}$ is a concrete value belonging to ($-inf, +inf$). Next, to ease the following calculations, we convert the real-value $\varOmega _{i, j}$ into a Boolean-value $\varPsi _{i, j}$, which is formulated by Eq. (6). In Eq. (6), $\varPsi _{i, j}$ value is mapped to be 1 or 0, whose rationale is explained as follows: let us consider a data point *D* and a hyperplane *H*; if point *D* is above the hyperplane *H*, then the $\varPsi _{i, j}$ value corresponding to *D* is equal to 1; otherwise, if point *D* is below the hyperplane *H*, then the $\varPsi _{i, j}$ value corresponding to *D* is equal to 0. This way, we can use such a kind of position relationship between point *D* and hyperplane *H* to evaluate whether two points are close or not. This is the theoretical basis behind the hash mapping operation adopted in Eq. (6).

This way, we convert the *K*-dimensional vector $$A_{i,j}$$ in Eq. (2) into a Boolean-value $$\varPsi _{i, j}$$. Correspondingly, the $$N*M*K$$ tensor $$\kappa$$ in Eq. (1) can be simplified to be the $$N*M$$ matrix $$\kappa$$ in Eq. (7). Next, we continue to simplify the $$N*M$$ matrix $$\kappa$$ into an *N*-dimensional vector, which could be finished by the transformation in Eq. (8). Here, $$\pi _{i}$$ is the decimal value corresponding to the Boolean vector ($$\varPsi _{i, 1}$$,..., $$\varPsi _{i, M}$$). For example, if ($$\varPsi _{i, 1}$$,..., $$\varPsi _{i, M}$$) = (1, 1, 1), then $$\pi _{i}$$ = 7. This way, we successfully convert the $$N*M$$ matrix $$\kappa$$ in Eq. (7) into an *N*-dimensional vector $$\kappa$$ in Eq. (8). In other words, each student $$s_{i}$$ is corresponding to a concrete decimal value $$\pi _{i}$$. According to the index theory, decimal value $$\pi _{i}$$ can be considered as the health index of student $$s_{i}$$.

6 $$\begin{aligned} \varPsi _{i, j}=\left\{ \begin{array}{rcl} 1 &{} &{} \text {when}\ \varOmega _{i, j}>0\\ 0 &{} &{} \text {when}\ \varOmega _{i, j}<0 \end{array} \right. \end{aligned}$$

7 $$\begin{aligned} \kappa = \begin{array}{cc} &{} c_{1}\quad \cdots \quad c_{M}\\ \begin{array}{c} s_{1} \\ \vdots \\ s_{N} \end{array} &{} \left[ \begin{array}{ccc} \varPsi _{1, 1} &{} \cdots &{} \varPsi _{1, M} \\ \vdots &{}\ddots &{}\vdots \\ \varPsi _{N, 1} &{} \cdots &{} \varPsi _{N, M} \end{array}\right] \end{array} \end{aligned}$$

8 $$\begin{aligned} \kappa = \begin{array}{cc} \begin{array}{c} s_{1} \\ \vdots \\ s_{N} \end{array} &{} \left[ \begin{array}{c} \pi _{1} \\ \vdots \\ \pi _{N} \end{array}\right] \end{array} \end{aligned}$$

The advantages of health index here are three-fold: first, health index contains little privacy of students and hence can be transmitted or released to the cloud platform with less privacy risks, which can minimize the privacy disclosure concerns of people when a cloud platform integrates the distributed data of people together for uniform data processing and mining; second, health index-based similar student retrieval is rather quick; third, health index-based similar student retrieval results are rather close to the similar student retrieval results based on original health data that are sensitive to students. Therefore, we use the health indexes of students to take part in the subsequent distance calculation (Step 2) and anomaly detection (Step 3). This way, we can guarantee that the distance calculation and anomaly detection process is time-efficient and privacy-guaranteed.

### Step 2: Calculate the similarity between each pair of students based on their health indexes

As discussed in Step 1, each student $s_{i}$ is corresponding to a concrete decimal value $\pi _{i}$. Here, $\pi _{i}$ is obtained from the random vector *B* in Eq. (3) which bring additional uncertainty in creating the accurate health indexes of students. To minimize the uncertainty, *q* (*q* is an integer larger than 1) decimal values are necessary to be obtained for each student $s_{i}$. In concrete, for each $s_{i}$, we repeat the operations in Eqs. (3)-(8) *q* times to generate $\pi _{i, 1}$,..., $\pi _{i, q}$. After that, we get a new matrix $\kappa$ as specified in Eq. (9). According to Eq. (9), each student $s_{i}$ is corresponding to a *q*-dimensional vector ($\pi _{i, 1}$,..., $\pi _{i, q}$). Then vector ($\pi _{i, 1}$,..., $\pi _{i, q}$) can be regarded as the health index of student $s_{i}$.

9 $$\begin{aligned} \kappa = \begin{array}{cc} \begin{array}{c} s_{1} \\ \vdots \\ s_{N} \end{array} &{} \left[ \begin{array}{c} \left( \pi _{1, 1} \cdots \pi _{1, q} \right) \\ \vdots \\ \left( \pi _{N,1} \cdots \pi _{N, q} \right) \end{array}\right] \end{array} \end{aligned}$$

With the health indexes of two students $s_{i}$ and $s_{j}$, i.e., ($\pi _{i, 1}$,..., $\pi _{i, q}$) and ($\pi _{j, 1}$,..., $\pi _{j, q}$), we can compute the similarity between $s_{i}$ and $s_{j}$ (denoted by $Sim(s_{i}, s_{j})$) based on the formula in Eqs. (10)-(11). Here, $Sim(s_{i}, s_{j})$ represents the number of dimensions whose values of $s_{i}$ and $s_{j}$ are equal. For example, let us consider two students $s_{1}$ and $s_{2}$ whose health indexes are (1, 2, 3, 4, 5) and (1, 2, 3, 6, 7), respectively. Then their similarity $Sim(s_{1}, s_{2})$ = 3 according to Eqs. (10)-(11). Furthermore, to loosen the judgement condition in Eq. (11), we create *p* (*p* is an integer larger than 1) hash tables, i.e., we generate $\kappa _{1}$ ,..., $\kappa _{p}$ by Eq. (9). Next, we update Eq. (11) to be Eq. (12) where the similarity judgement condition is loosened considerably.

10 $$\begin{aligned} Sim(s_{i}, s_{j}) = \sum \limits _{z=1}^{q} Sim_{i, j, z} \end{aligned}$$

11 $$\begin{aligned} \begin{aligned} Sim_{i, j, z} = 1, \text {iff}\ \pi _{i, z} = \pi _{j, z}(z=1, 2,\ldots , q) \end{aligned} \end{aligned}$$

12 $$\begin{aligned} \begin{aligned} Sim_{i, j, z} = 1, \text {iff}\ \pi _{i, z} = \pi _{j, z}(z=1, 2,\ldots , q) \\ \text {holds}\ \text {in}\ \text {any}\ \kappa _{1},\ldots , \kappa _{p} \end{aligned} \end{aligned}$$

### Step 3: Student health condition clustering and anomaly detection

According to the similarity between different students calculated in Step 2, we can cluster the students into different groups. In general, the students whose similarity with each other is large belong to an identical group. For example, if two students whose similarity is *q*, then they would be put into an identical group. Here, for discovering the most similar students, we set a threshold $T (T \le q)$ for $Sim(s_{i}, s_{j})$. More specifically, only the students $s_{i}$ and $s_{j}$ whose $Sim(s_{i}, s_{j})$ is not smaller than *T* are deemed as similar. Following such a clustering rule, we can divide all the students into different groups. Furthermore, the students who have no similar students could be regarded as anomaly. This way, we can recognize the anomaly students accurately and meanwhile the sensitive information contained in health data transmitted to the cloud platform can be protected very well.

Next, we use the following algorithm to better ease the understanding of our Ano-Det method.
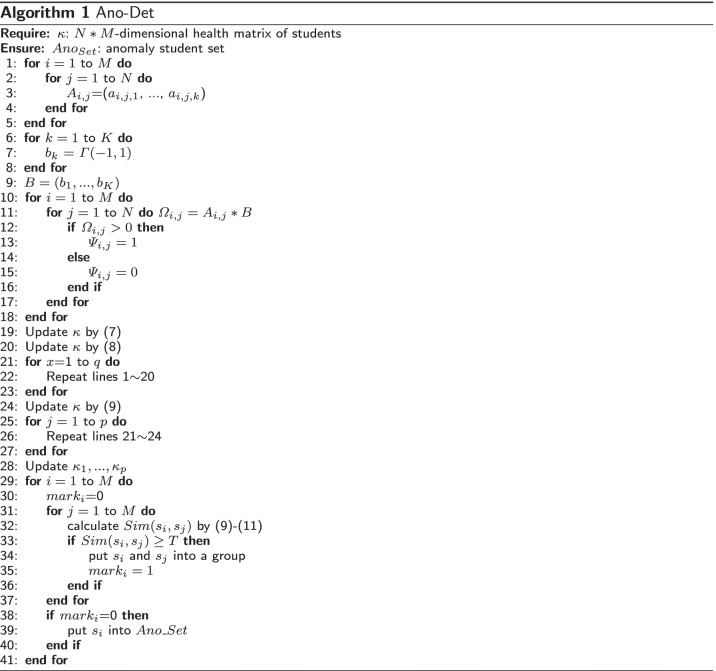


## Evaluation

We evaluate the feasibility of Ano-Det method via a set of simulate experiments which deployed on WS-DREAM dataset. In concrete, the users and services in the dataset are used to simulate the students and health criteria involved in Ano-Det method. Moreover, only one dimension of response time in the dataset is considered [8]. For comparisons, we also compare Ano-Det method with existing methods $\text {SerRec}_{\text {distri-LSH}}$ [45] and UCF (user-based collaborative filtering). The experiments are run on a computer with 3.20 GHz processor and 8.0 GB memory. The algorithm is developed by Windows 7 and Python 2.7. In concrete, the following three profiles are investigated to prove the algorithm performances.

**Fig. 2:**
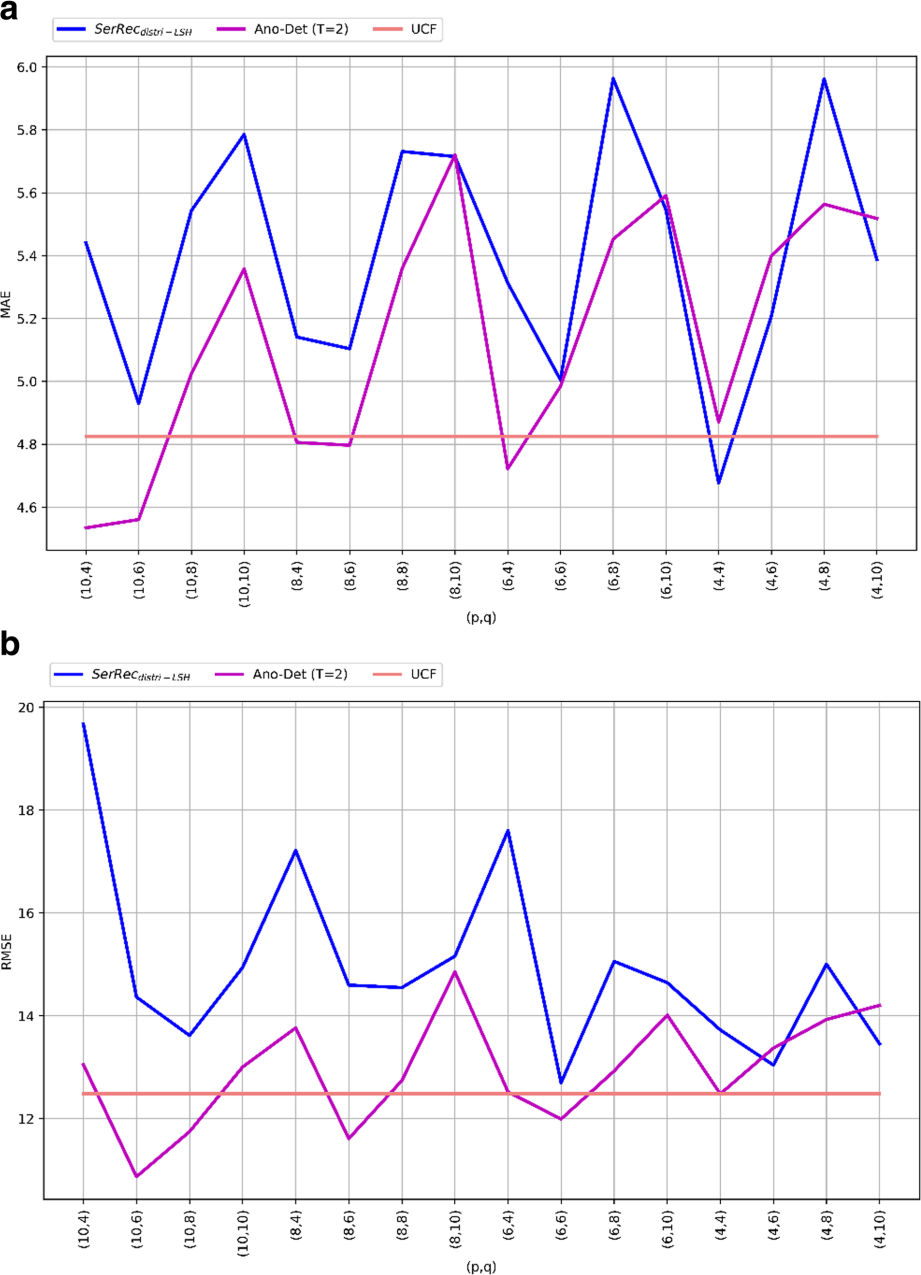
Anomaly detection accuracy of three methods

### Profile 1: detection accuracy of three methods

Here, the anomaly detection accuracy of Ano-Det method is measured and compared to $\text {SerRec}_{\text {distri-LSH}}$ and UCF. Here, the accuracy is reflected by MAE and RMSE. In the parameter settings, student volume *N* = 142, health criteria volume *M* = 4500, time point volume *K* = 64, threshold *T* = 2, *p* and *q* are both varied from 4 to 10. Experimental results are presented in Fig. 2. Concretely, MAE comparison is presented in Fig. 2a where Ano-Det method performs better than SerRecdistri-LSH method (i.e., the MAE of Ano-Det is smaller than $\text {SerRec}_{\text {distri-LSH}}$) because time factor is considered in Ano-Det method and therefore, more accurate anomaly detection results are guaranteed. Although the accuracy of Ano-Det method is worse than UCF method (i.e., the MAE of Ano-Det is larger than UCF), Ano-Det method can secure user privacy well while UCF method cannot. In summary, the comparison results shown in Fig. 2 mean that our Ano-Det method can achieve a good balance between anomaly detection accuracy and privacy-preservation capability. Besides, RMSE comparison is presented in Fig. 2b where similar results are observed as in Fig. 2a. The reason is the same as that analyzed in Fig. 2a and will not be repeated again.

### Profile 2: detection efficiency of three methods

**Fig. 3:**
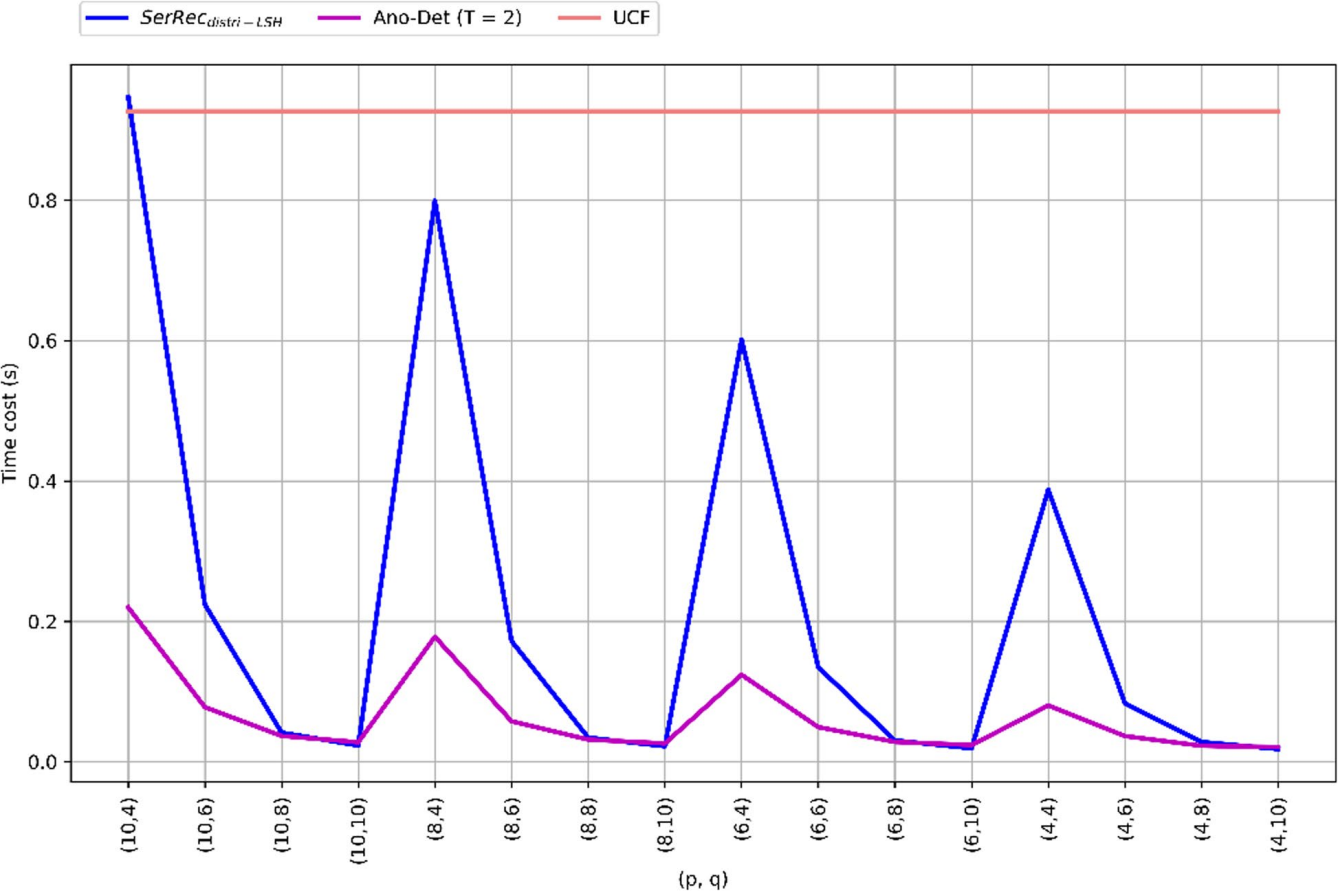
Anomaly detection efficiency of three methods

Here, the anomaly detection efficiency of Ano-Det method is compared to $\text {SerRec}_{distri-LSH}$ and UCF. Here, the efficiency is measured by time cost. In the parameter settings, student volume *N* = 142, health criteria volume *M* = 4500, time point volume *K* = 64, threshold *T* = 2, *p* and *q* are both varied from 4 to 10. Comparison results are demonstrated in Fig. 3. As shown in Fig. 3, Ano-Det and $\text {SerRec}_{\text {distri-LSH}}$ consume less time than the baseline UCF method, because the former two methods both use index technique which is often time efficient in big data context while UCF does not. Furthermore, Ano-Det consumes less time than $\text {SerRec}_{\text {distri-LSH}}$ because time factor is considered in Ano-Det and therefore, less but more similar students could be obtained in Ano-Det. Correspondingly, the time consumed in anomaly detection phase is reduced considerably. Another obvious observation is available from Fig. 3: the time costs of Ano-Det and $\text {SerRec}_{\text {distri-LSH}}$ both decline with the growth of q and the drop of p. The reason can be analyzed as follows: a larger q and a smaller p both mean more rigid similarity judgment conditions according to Eqs. (9)-(12); in this situation, only fewer similar students are returned for clustering and anomaly detection. Therefore, the time cost is decreased accordingly. In summary, the time cost of Ano-Det method is relatively small and hence can be applicable to big data analysis scenarios where a quick response is often necessary.

### Profile 3: detection accuracy of Ano-Det w.r.t. (*p*, *q*, *T*)

**Fig. 4:**
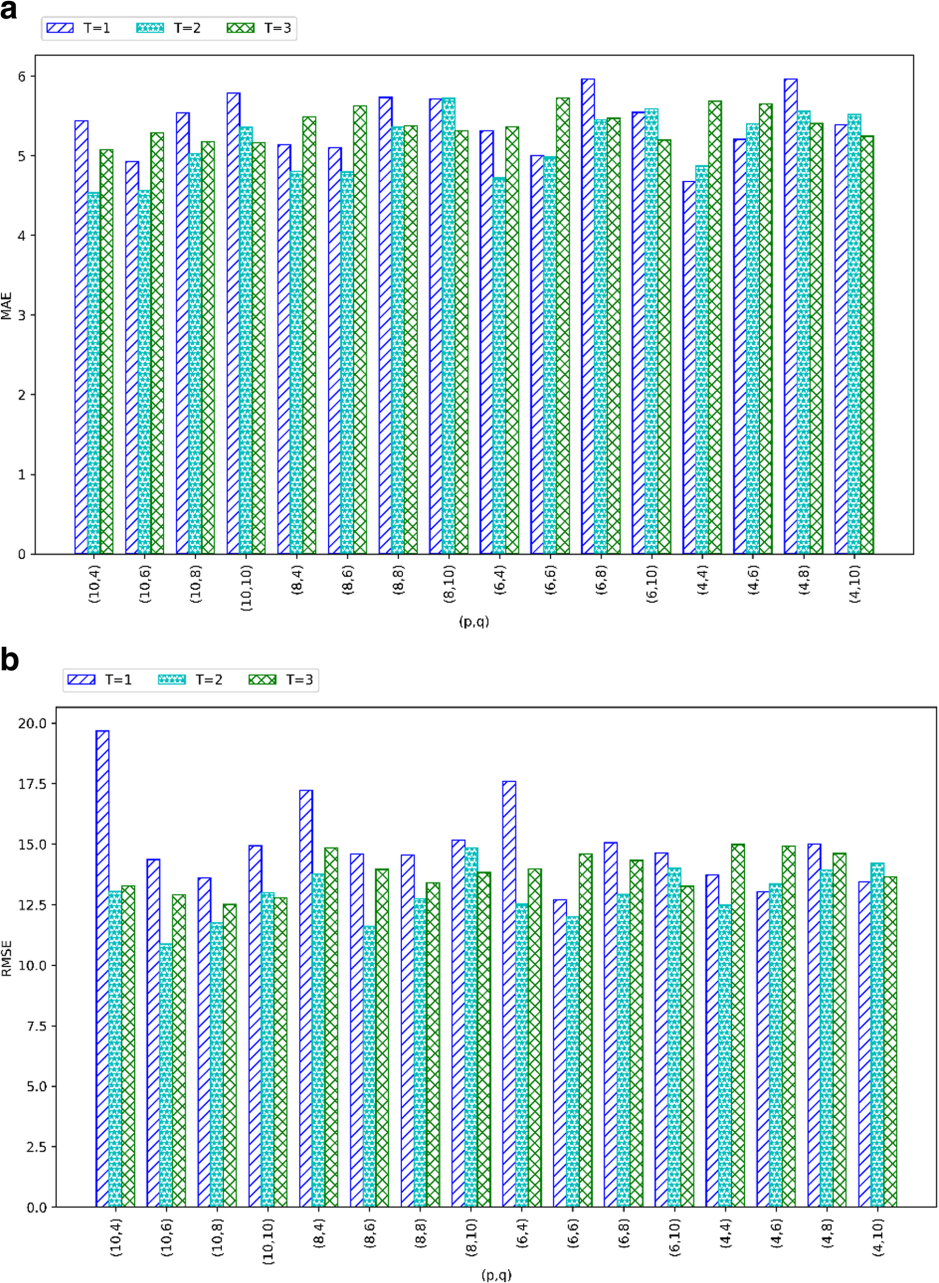
Anomaly detection accuracy of Ano-Det w.r.t. (*p*, *q*, *T*)

According to the algorithm analysis of Ano-Det method, three important parameters are existent including q in Eq. (9), *p* in Eq. (12) and threshold *T* in Step 3. Through analyzing Algorithm 1, we can find that these three factors are all related to the similarity calculation as well as the subsequent student clustering and anomaly detection. Therefore, we design a set of experiments in this profile to observe the relationship of Ano-Det’s performances (e.g., MAE and RMSE) with respect to the three parameters. In the parameter settings, student volume *N* = 142, health criteria volume *M* = 4500, time point volume *K* = 64, threshold *T* is varied from 1 to 3, *p* and *q* are both varied from 4 to 10. Comparison results are demonstrated in Fig. 4. As shown in Fig. 4a, the MAE of Ano-Det method is often the largest when *T* = 1. This can be explained as follows: *T* = 1 means that two students are similar as long as their indexes are equal in terms of any of the *q* dimensions (in Eq. (9)) in any of *p* hash tables (in Eq. (12)). The above similarity evaluation condition is relatively looser compared to the conditions corresponding to *T* = 2 and *T* = 3. As a consequence, more similar students are returned for subsequent student clustering and anomaly detection even the returned similar students are actually not very similar with each other. Therefore, the anomaly detection accuracy is decreased more or less. Similar results can be obtained from Fig. 4b whose reason is the same as that in Fig. 4a.

## Conclusions

It has become a key research issue to guarantee the healthy living of people especially the students who are of relative weak physique. In this situation, precise recognition of the anomaly in student health conditions is beneficial to the quick discovery of potential patients. Fortunately, the quick development of mobile cloud computing technologies [46] and wearable sensors has provided a promising way to monitor the real-time health conditions of students and find out the anomalies timely. However, two challenges are present in the above anomaly detection issue. First, the health data monitored by massive wearable sensors and transmitted to the cloud platform are often massive and updated frequently, which probably leads to low efficiency of anomaly detection. Second, the health data of students are often sensitive enough, which probably impedes the integration of health data in cloud platform. In view of these challenges, a time-efficient and privacy-aware anomaly detection solution for students is proposed with wearable sensors in mobile cloud computing. Finally, we prove the feasibility of our research proposal via a set of simulated experiments.

In this paper, we only discuss the health data with identical formats. However, data format variety is one of the key characteristics of big data applications [47–52]. Therefore, we will further improve our anomaly detection algorithm by accommodating the diversity of health data types or formats in future. In addition, energy saving is an important issue to tackle the challenge raised by big data [36, 53–56]; therefore, we will consider to introduce some effective energy saving techniques into our proposal in future.

## Data Availability

WS-DREAM: http://wsdream.github.io/.

## Abbreviations

CNN: Convolutional Neural Networks
GRU: Gate Recurrent Unit
MSD: Markov square distance
SHM: Structured Health Data
